# Depression recognition using voice-based pre-training model

**DOI:** 10.1038/s41598-024-63556-0

**Published:** 2024-06-03

**Authors:** Xiangsheng Huang, Fang Wang, Yuan Gao, Yilong Liao, Wenjing Zhang, Li Zhang, Zhenrong Xu

**Affiliations:** School of Biomedical Engineering, South-Central Minzu University, No.182, Minzu Avenue, Hongshan District, Wuhan City, 430074 Hubei Province China

**Keywords:** Depression, Pre-training model, Voice features, Wav2vec 2.0, DAIC-WOZ, Biomedical engineering, Data mining

## Abstract

The early screening of depression is highly beneficial for patients to obtain better diagnosis and treatment. While the effectiveness of utilizing voice data for depression detection has been demonstrated, the issue of insufficient dataset size remains unresolved. Therefore, we propose an artificial intelligence method to effectively identify depression. The wav2vec 2.0 voice-based pre-training model was used as a feature extractor to automatically extract high-quality voice features from raw audio. Additionally, a small fine-tuning network was used as a classification model to output depression classification results. Subsequently, the proposed model was fine-tuned on the DAIC-WOZ dataset and achieved excellent classification results. Notably, the model demonstrated outstanding performance in binary classification, attaining an accuracy of 0.9649 and an RMSE of 0.1875 on the test set. Similarly, impressive results were obtained in multi-classification, with an accuracy of 0.9481 and an RMSE of 0.3810. The wav2vec 2.0 model was first used for depression recognition and showed strong generalization ability. The method is simple, practical, and applicable, which can assist doctors in the early screening of depression.

## Introduction

Depression, a prevalent mental disorder, is characterized by impaired emotional regulation, persistent low mood, reduced interest or pleasure, impaired concentration, and, in some cases, suicidal contemplation^1^. It affects over 322 million people worldwide and shows an alarming upward trend in prevalence^2^. The World Health Organization (WHO) has identified it as the fourth leading cause of disability, with predictions indicating that it could rise to the second position by 2030^3^. The traditional methods for assessing psychiatric pathology mainly rely on subjective evaluations, such as patients’ verbal descriptions, reports from familiar individuals about their behaviors and experiences, and observations of mental states^4^. And it typically requires a wealth of professional knowledge as support.

Fortunately, artificial intelligence technology can utilize big data and intelligent algorithms to provide earlier diagnosis and intervention for the treatment of depression. Numerous methods have been proposed, encompassing biomarker-based approaches^5^, social media data-based techniques^6,7^, video-based methodologies^8,9^, audio-based methods^10–13^, electroencephalogram(EEG)-based approaches^14,15^, galvanic skin-based techniques^16,17^, and multimodal fusion strategies^18–21^. Within these approaches, there are invasive and non-invasive approaches. Non-contact diagnostic methods have clear advantages in ensuring patient safety, enhancing convenience, and reducing unnecessary interventions. For instance, Mustafa et al. used non-intrusive RF sensing for early diagnosis of spinal curvature syndrome disorders^22^. As a non-invasive diagnostic approach, voice signals are widely utilized for the detection of emotional disorders, owing to their rich abundance of pathophysiological information^23–25^. Moreover, employing the human voice for automatic diagnostic models in depression offers several advantages, including non-invasive data acquisition, relatively straightforward data collection, and low recording costs. However, the current approach of using machine learning methods for voice-based diagnostics often shows suboptimal recognition accuracy. Although deep learning methods have shown promising results, they still face challenges such as the scarcity of large-scale, high-quality datasets, the reliance on hand-designed features that demand domain expertise, and the time-consuming and subjective nature of feature engineering.

In response to the limitations of hand-designed features, Wang et al. have developed a generalized framework for robust feature extraction based on deep learning methods^26^. In this study, we propose the utilization of a voice-based pre-trained model to automatically extract voice features. Pre-trained models have been trained on large-scale data, exhibiting strong feature extraction capabilities and excellent generalization performance. As a result, they demonstrate high performance in various downstream tasks^27^. Previous studies encountered challenges in directly processing raw audio data, resulting in high computational costs and limiting model optimization. However, the introduction of wav2vec 2.0, a state-of-the-art technique for audio representation and transfer learning, has brought significant advancements in raw audio processing. It can be fine-tuned with limited annotated data to achieve, and in some case even surpass, the original best performance^28^. The wav2vec 2.0 model comprises a feature encoder, transformer, and quantization module. It is specifically designed to learn comprehensive representation from raw audio data, eliminating the need for large amounts of labeled data. The feature encoder is capable of extracting various levels of features from the raw audio signal. The transformer excels at capturing global voice characteristics. Additionally, the quantization module efficiently quantifies voice features, reducing the number of model parameters without compromising performance. The structure design enhances the model’s compactness and efficiency. Consequently, this research direction shows great promise in the field of depression detection.

In this study, the wav2vec 2.0 model was employed to extract high-quality voice features, thereby enhancing recognition accuracy within a small sample dataset. A compact fine-tuning network was connected to the output of the wav2vec 2.0 model for obtaining classification results. The proposed model was fine-tuned on the DAIC-WOZ dataset. Unlike traditional machine learning or deep learning method, which require tedious manual feature extraction, the proposed method automatically selects representational features for training. Experiments results obtained from the DAIC-WOZ dataset demonstrated the effectiveness of this approach.

To the best of our knowledge, this work represents the first attempt to utilize the wav2vec 2.0 model to enhance the depression recognition accuracy. Our contributions are summarized as follows:

1. It outlines the current research in depression recognition, discusses limitations, and suggests future research directions.
2. Proposing an artificial intelligence method for effectively recognizing depression through voice signals to enhance diagnostic accuracy and treatment efficiency.
3. The proposed method utilizes the wav2vec 2.0 pre-trained model to extract high-quality speech features, streamlining feature extraction and reducing the reliance on manually crafted features.
4. Demonstrates that excellent performance can be achieved using solely voice data, eliminating the need for complex multimodal data and mitigating the risk of personal privacy breaches.

The structure of the paper is organized as follows: “Related work of depression recognition” section provides an overview of the research on automated detection of depression. “Material and methods” section introduces the dataset, describes the depression recognition model, and provides details about the experimental setup. “Result and discussion” section analyzes the experimental results and presents a discussion of the findings. “Limitations” section addresses potential biases and discusses any challenges encountered during the experiment. “Conclusions” section summarizes the paper and outlines the future work plan.

## Related work of depression recognition

In recent years, various indicators have been explored for automated depression detection. Wollenhaupt-Aguiar et al. used machine learning methods to obtain discriminative features based on biomarkers^5^. However, biomarkers are difficult to obtain and relatively expensive to analyze. Based on social media data, Zhou et al. employed a time-aware attention multimodal fusion network (TAMFN) for depression detection^6^. Li et al. utilized CNN Asynchronous Federated optimization (CAFed) for depression detection^7^. Based on visual cues, Guo et al. utilized temporal dilated convolutional network (TDCN) for depression detection^8^. Casado et al. utilized remote photoplethysmography of facial video to identify depression^9^. Despite the rich information contained in the video, factors like lighting, weather, camera movement, etc., can impact the results. Ksibi et al. used EEG data to build three classification models based on extreme gradient boosting (XGBoost), random forest (RF), and one-dimensional convolutional neural network (1D CNN) for depression detection^14^. Wang et al. used AlexNet network to train EEG signals for depression detection^15^. However, the optimization of spatial information in multichannel EEG data remains a challenge, and compressing high-dimensional data can easily lead to information loss. In the field of electrodermal research, Sharma et al. employed an autoencoder network (AEN) and deep neural networks (DNN) to detect depressive states^16^, and Lyu et al. developed a discriminative model using the support vector machine (SVM) algorithm^17^. These approaches typically require specialized testing equipment and proper skin contact. The heightened activity of sweat glands in warm or humid conditions often impacts signal accuracy. Motion artefacts are commonly present in the electrodermal signals, affecting the classification results.

In addition, multimodal models are also one of the hotspots of research. Qayyum et al. combined audio spectrograms and multiple frequencies of EEG signals to improve the diagnostic performance^18^. Fang et al. and Xia et al. use three modalities, namely video, audio, and text, for their respective studies. However, there are difference in their approaches. Fang et al. proposed a multimodal fusion model with multi-level attention mechanism (MFM-Att) for depression detection, incorporating a multilevel attention mechanism^19^. On the other hand, Xia et al. pre-fused the three modalities before feeding them into a bidirectional long short-term memory (Bi-LSTM) network to establish a multimodal fusion representation. Subsequently, this multimodal fusion representation was input into a graph neural network (GNN) for depression detection^20^. Nevertheless, it is important to note that multimodal models entail a complex processing flow and necessitate simultaneous handling of information from various data sources, resulting in high computational complexity. Additionally, data collection and processing for multimodal models can be challenging.

Depressed individuals exhibit distinct acoustic characteristics compared to non-depressed individuals, and the effectiveness of using voice for depression detection has been demonstrated^29^. Exploration of depression detection based on voice signals began with the widespread adoption of two key approaches: traditional machine learning and neural networks. Gao et al. used Random Forest Algorithm^30^ and Shi et al. used SVM for depression recognition^31^. Aharonson et al. also chose machine learning methods to automatically categorize depression severity^32^. Depression detection studies based on machine learning methods usually struggle to learn meaningful knowledge representations, and classification accuracy relies heavily on feature selection. With the development of technology, many studies have begun to apply deep learning methods to automated depression diagnosis. Previous research has focused more on the use of handmade acoustic features. Rejaibi et al. combined hand-crafted features with deep learning to improve depression recognition performance^33^. Liu et al. used mel frequency cepstral coefficient (MFCC) features as input to convolutional-bidirectional long short-term memory (CNN-BLSTM) for depression detection^34^.

Although traditional acoustic features perform well in the field of voice recognition, they are not tailored for the task of depression recognition, and the knowledge representation of depression is still insufficient. Hence, people have turned to mining deep learning features for depression detection. Miao et al. combined voice features to classify depression, and the accuracy reached 0.85^35^. Zhao et al. used frame-level features to capture time information of depressed voice as input to the long-short term memory (LSTM) layer and achieved an average accuracy of 0.902^36^. Yang et al. developed the end-to-end learning framework with attention guided learnable time-domain filterbanks (DALF) module, which is designed to generate task relevant spectral features^10^. Sardari et al. used a convolutional autoencoder (CNN AE) to extract highly correlated and compact set of features^11^. Sun et al. proposed a model combining unsupervised encoding with transformers to achieve depression detection^12^. Yin et al. utilized transformers and parallel convolutional neural networks (parallel-CNN) to extract valuable information with acceptable complexity^13^. Yang et al. designed the deep convolutional generative adversarial network (DCGAN) feature augmenting network for improved depression severity estimation^37^. Srimadhur et al. used an end-to-end convolutional neural networks (CNN) model to detect depression^38^. Zhang et al. proposed a multilevel depression state detection method based on fine-grained cue learning^39^. However, most of the deep learning-based methods have complex model structures and usually require a large amount of data training to improve the accuracy.

In this section, we summarize the work related to automated detection of depression, details of which can be viewed in Table 1.

**Table 1:** Summary of relevant work on depression detection.

References	Analyzed	Technique used	Results	Limitation
^5^	Interleukin-4, Interleukin-10, et al	SVM	Sensitivity: 62.00%, specificity: 66.00%	Biomarkers are difficult to obtain and relatively expensive to analyze
^6^	Social media data (D-Vlog)	TAMFN	Precision:66.02%, F1-score:65.82%	Social media data contains false information
^7^	Social media data (Weibo)	CAFed	Accuracy:86.67%	Social media data contains false information
^8^	Video	TDCN	Accuracy:85.70%	Susceptible to environmental factors
^10^	Audio	DALF	F1 is 78.4% on the DAIC-WOZ dataset	The model structure is complex and the model performance is not optimal
^11^	Audio	CNN AE	Precision:71.00%	The model performance is not optimal
^12^	Audio	An unsupervised autoencoder network	MAE:4.01, RMSE:5.96, F1-score:87.00%	Not directly analyzing the raw speech signal. The model performance is not optimal
^13^	Audio	Transformer + Parallel-CNN	Precision:95.00%, recall:92.20%, F1-score:93.60%,	The model structure is complex. Not directly analyzing the raw speech signal
^15^	EEG	AlexNet	Partial channel accuracy greater than 70.00%	Insufficient use of spatial information in multichannel data. Model performance is susceptible to individual differences
^16^	Electrodermal	AEN + DNN	94.0% accuracy with AEN	High threshold of detection technology. The reading of signals is easily disturbed
^17^	Electrodermal	SVM	Accuracy: 78.00%, sensitivity:78%, specificity:82%	Difficulty in learning meaningful representations. Model classification performance is not performing well enough
^18^	Audio + EEG	Vision Transformer	Precision:97.20%, recall:97.30%	Multimodal data processing is cumbersome. Data collection is difficult
^19^	Audio + Video + Text	MFM-Att	MAE:3.18, RMSE:3.68	
^20^	Audio + Video + Text	Bi-LSTM + GNN	Accuracy:73.65%	

## Material and methods

### Dataset and preprocessing

#### Experimental dataset

The data used in this paper comes from the DAIC-WOZ dataset, which is part of a larger clinical interview corpus called the Distress Analysis Interview Corpus (DAIC)^40^. This dataset also uses a virtual human interviewer, and each audio file contains dialogue data between a patient and a virtual agent named Ellie with a sampling rate of 16 kHz. The dataset provides 189 audio files that has been divided into training, validation, and test sets. The audio ID numbers range from 300 to 492, excluding 342, 394, 398, 460 due to technical reasons. The dataset includes information such as patient ID, binary labels, and PHQ-8 scores. For more details, please see Table 2.

**Table 2:** Relevant statistical information on the DAIC-WOZ dataset.

Items	Data	Items	Data
Total samples	189	Train set	107
Male	102	Validation set	35
Female	87	Test set	47
Non-depressed	133	Sampling frequency	16 kHz
Depressed	56		

**Figure 1:**
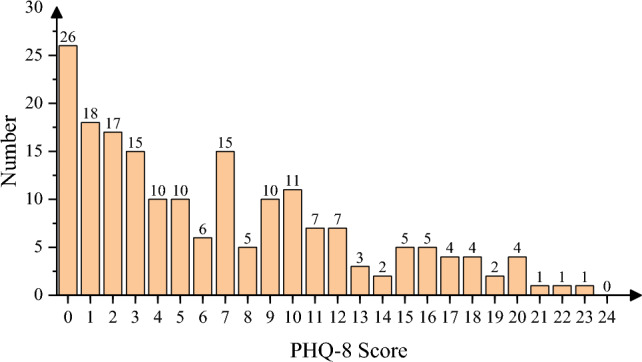
Sample distribution of PHQ-8 score.

The sample distribution of PHQ-8 scores is shown in Fig. 1.

#### Preprocessing

The audio files in the DAIC-WOZ dataset were preprocessed to enhance data quality. While wavelet transforms and signal denoising techniques are commonly employed for data processing^41^, given the objective of training a robust model in this study, we have employed a distinct strategy. Specifically, we employed voice segmentation and merging techniques to extract segments that exclusively contain patient voice.

The TRANSCRIPT files provided by the DAIC-WOZ dataset contain detailed records of the start and end times of each participant’s conversations. A total of 189 TRANSCRIPT files, each corresponding to a specific participant. To illustrate this, the excerpts extracted from these conversation records are shown in Table 3.

**Table 3:** Extracts from the transcripts of the conversations.

Start time (s)	Stop time (s)	Speaker	Value
49.256	50.406	Ellie	How are you doing today
50.686	51.836	Participant	Okay how ‘bout yourself
52.576	54.136	Ellie	I’m great thanks
54.816	56.236	Ellie	Where are you from originally
56.586	57.996	Participant	Here in california
58.216	58.936	Ellie	Really
59.066	59.756	Participant	Yeah
60.526	62.936	Ellie	What are some things you really like about l_a
63.396	67.696	Participant	Oh well that it’s big and broad there’s a lot to do a lot of um
69.416	72.266	Participant	Um job opportunities than other states
72.766	77.626	Participant	Um pretty much that it’s big and there’s a lot you can do here
79.056	79.866	Ellie	Yeah

Specifically, first, based on the start and end times of each patient’s utterance recorded in the transcript files of the DAIC-WOZ dataset, 189 raw audio files were segmented into independent segments, each containing only one sentence spoken by the patient. This process resulted in over thirty thousand small segments. We merged these segments in sequential order, grouping every five voice segments together. This procedure generated a total of 6545 new audio files. It is important to note that before merging voices, it must be determined that only voice data from the same patient ID can be merged.

**Figure 2:**
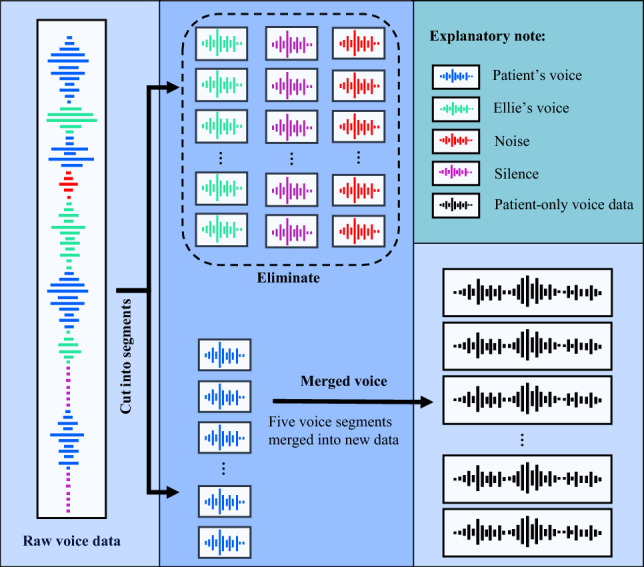
Voice data preprocessing steps.

At the same time, Ellie’s voice and long periods of silence were excluded. This process resulted in obtaining patient-only voice data, as depicted in Fig. 2, which outlines the steps involved in voice data preprocessing.

#### Redivision of dataset

Adequate data samples, obtained after preprocessing, have helped alleviate the issue of sample imbalance. Therefore, data augmentation is no longer necessary. Subsequently, the preprocessed dataset was randomly divided into training set, validation set, and test set according to the ratio of 6:2:2. The relevant information about the dataset after preprocessing and redivision is summarized in Table 4.

**Table 4:** Relevant statistical information in preprocessed DAIC-WOZ dataset.

Items	Data	Items	Data
Total samples	6545	Ndep	4513
Male speaks	3484	Dep	2032
Female speaks	3061	Non	2861
Train set	3927	Mild	1597
Validation set	1309	Moderate	1102
Test set	1309	Severe	985

The training set and validation set were used to fine-tune and evaluate the model performance, and the test set was used to evaluate the model reliability in actual applications. To ensure a balanced proportion of labels, the four categories of non, mild, moderate, and severe were separated before being randomly grouped. For repeatability purposes, we used a random seed for division and set it uniformly to 103.

### Model and experimental setup

#### Voice recognition model

The combination of pre-training and fine-tuning has proven to be an effective learning method^42^. During pre-training, a model is trained on a large dataset in an unsupervised manner to learn meaningful representations. This approach prevents over-fitting to task-specific data because the pre-training stage acts as a regularizer, providing a wealth of prior information.

**Figure 3:**
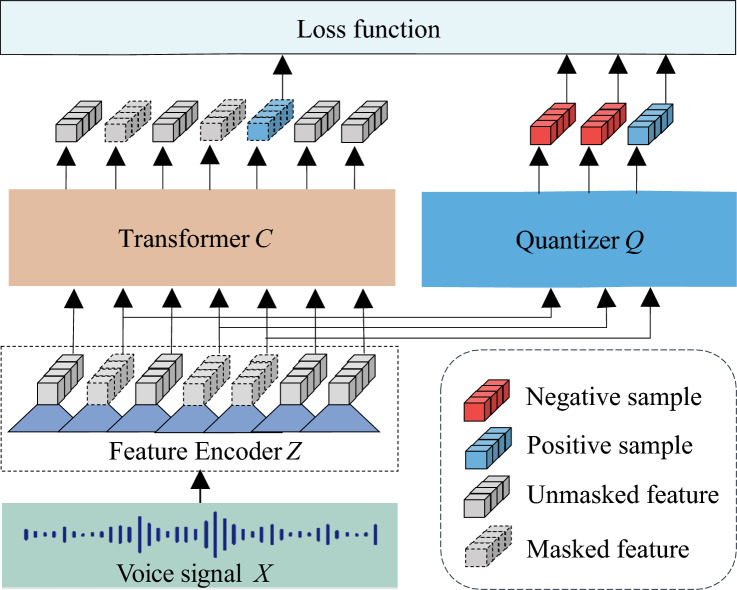
Framework of the wav2vec 2.0 pre-trained model.

Wav2vec 2.0 voice pre-training model, with its strong potential in voice processing, can learn discrete voice units and end-to-end context representation. It has evolved from previous models such as contrastive predictive coding (CPC), Wav2vec, and Vq-Wav2vec. As described by Baevski et al.^28^, it consists of a feature encoder module, a quantization module, and a transformer module, as shown in Fig. 3. The automatic extraction of voice features by the wav2vec 2.0 model proceeds as follows.

First, the feature encoder maps the raw audio $X$ to latent voice representations $Z:\left\{ {z_{1} ,z_{2} , \cdots ,z_{n} } \right\}$, represented as $f:X \to Z$. Specifically, the raw audio $X$ was input into multiple convolution layers, where the first convolution layer was a time convolution and a GELU activation function was adopted. A group normalization method was used prior to GELU to normalize each output channel of the first layer. In each layer, the output data has been normalized to improve robustness. At the last layer, an L2 regularization term was applied to the activation of the feature encoder to stabilize training. By utilizing multiple convolution layers, the feature encoder was able to acquire high-level semantic information from voice.

Second, in the quantization module, the output of the feature encoder $Z$ was discretized into a set of quantized voice features $Q:\left\{ {q_{1} ,q_{2} , \cdots ,q_{n} } \right\}$ that can be expressed as $h:Z \to Q$. $Q$ was used as a self-supervised objective. The quantization process reduced the difficulty of predicting real voice features because the output of the feature encoder $Z$ was transformed into a finite set of voice representations. The Gumbel softmax was used in this module to solve the problem that the feature space was not derivable and cannot be back-propagated after being discretized.

Third, the latent voice representations $Z$ from the feature encoder module were directly input to the transformer module to create context representations $C:\left\{ {c_{1} ,c{}_{2}, \cdots ,c_{n} } \right\}$. This process can be expressed as:$g:Z \to C$. Before being input to the transformer module, approximately half of the latent voice representations $Z$ were masked by the transformer’s encoder. This masking process brings the context representation $c_{i}$ of masked locations closer to the corresponding discrete features $q_{i}$. Due to self-attention mechanisms, each representation in the final output sequence may contain both local and global information^10^. The transformer module effectively captures long-distance dependencies and global context information, further improving the speech recognition performance of the model.

Finally, the quantized voice representations $q_{i}$ and context representations $c_{i}$ were used to calculate the loss function $L$. For each $c_{i}$ generated by the masked position, the positive example is $q_{i}$ generated by the quantization module at the same position, and the negative example is $\kappa$ quantization vectors $\tilde{q} \in Q_{{\text{t}}}$ generated by the quantization module at other masked positions. When the loss function $L$ reached its optimal value, high-quality voice features from the wav2vec2.0 model were input into a small fine-tuning network for voice depression recognition. The Loss function is defined as Eq. (1):

1 $$L = - \log \frac{{\exp (sim(c_{i} ,q_{i} ))/\kappa }}{{\sum\nolimits_{{\tilde{q}\sim Q_{{\text{t}}} }} {\exp (sim(c_{i} ,\tilde{q}))/\kappa } }}$$

There are two types of wav2vec2.0 models (Base and Large) that share the same encoder architecture but differ in the number of transformer blocks and model dimensions. The Base model is trained on the Librispeech corpus. It contains 7 convolutional layers and a 12-layer transformer structure. Each convolutional layer has 512 channels, with strides of (5, 2, 2, 2, 2, 2, 2) and kernel widths (10, 3, 3, 3, 3, 2, 2). The convergence of speed of the Base model is faster than that of the Large model, and the Base model requires fewer computational resources. Hence, the Base mode is more suitable for small datasets or scenarios with limited computing power.

**Figure 4:**
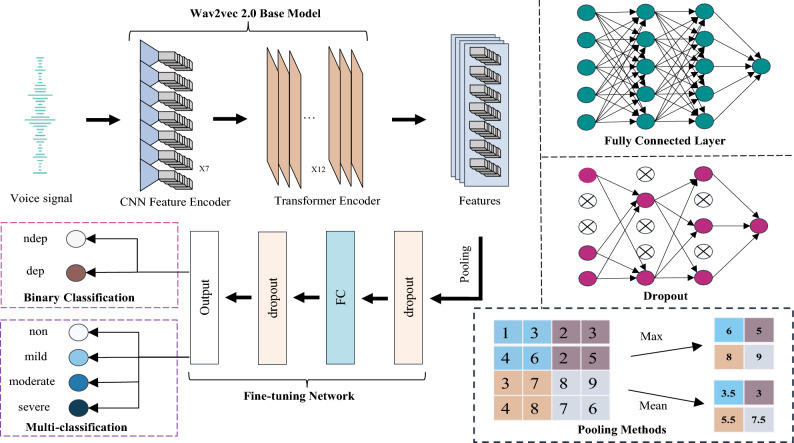
Overall framework of voice depression recognition.

A large amount of voice data has been used to train the wav2vec 2.0 pre-training model in advance, so it can quickly and accurately extract high-quality voice features. Furthermore, the DAIC-WOZ dataset was used to fine-tune the wav2vec 2.0 model for depression recognition. We performed all experiments using the Base model and connected a small fine-tuning network to its output for voice depression recognition as shown in Fig. 4.

As shown in Fig. 4, the voice signal was input into the wav2vec 2.0-base model, and the feature encoder module was used to capture the local information of the voice signal, while the transformer module was employed to capture global speech features, resulting in the extraction of high-quality voice features. These high-quality voice features were then pooled to expand the model’s receptive field, thereby enhancing both accuracy and robustness in voice recognition. Then, a dropout layer was applied in the small fine-tuning network to prevent over-fitting. By randomly dropping some neurons, it reduces co-adaptation between neurons so that the dependence of weight updating on fixed hidden nodes was decreased. The model proposed in this paper was fine-tuned through continuous iterations and the predictions of the model were output when the loss function reached the desired value.

#### Experimental tasks

Currently, research on depression predominantly revolves around binary classification, with limited exploration into multi-classification. Hence, this study has established two experimental tasks: binary classification and multi-classification.

In the binary classification task, we detected whether patients have the possibility of suffering from depression. The voice data labels had set to dep or ndep based on the binary values provided by the DAIC-WOZ dataset.

In the multi-classification task, the severity of depression was divided into four levels: no depression, mild depression, moderate depression, and severe depression. The PHQ-8 score (range 0–24) was discretized into 4 categories: [0–4], [5–9] , [10-14] and [15–24], and these four categories are labeled as non, mild, moderate and severe, respectively.

#### Parameter setting

Information about the experimental environment for this paper is as follows: CPU: 11th Gen Intel(R) Core(TM) i7-11700 @ 2.50 GHz; GPU: NVIDIA GeForce RTX 3090; RAM: 24G. Operating system: 64-bit Ubuntu 20.04.4 LTS; CUDA: 11.6; Python 3.7.

During the fine-tuning process, the pre-trained network parameters are also updated. To prevent large changes in parameters, a smaller learning rate should be chosen. Using a larger learning rate may cause drastic changes in the parameters that leads to bad performance of the model. This is because the pre-trained model has already been trained on a large amount of data and its parameters have been adjusted to an optimal state. We perform an experimental comparison of the effect of different learning rates ($1 \times 10^{ - 4}$, $1 \times 10^{ - 5}$, $1 \times 10^{ - 6}$) on model performance and find that the model performs best when the learning rate is $1 \times 10^{ - 5}$. Therefore, we fixed the learning rate at $1 \times 10^{ - 5}$ for all subsequent experiments. In addition, considering limited computational resources and the need to prevent memory overflow, the batch size was set to 4 for each run, with two gradient accumulations. As a result, the cumulative batch size was set to 8.

#### Evaluation methods

In the case of sample imbalance, relying solely on a single metric may lead to an incomplete evaluation. Therefore, the classification performance of the model was assessed using a comprehensive set of five evaluation metrics: accuracy, precision, recall, F1 score, and the RMSE. The formula for calculating accuracy is shown in Eq. (2)^43–45^:

2 $$Accuracy = \frac{TP + TN}{{TP + TN + FP + FN}}$$

The calculation formulas for *Precision*, *Recall*, and *F*1 score are shown in Eqs. (3)–(5)^46–48^:

3 $$Precision = \frac{TP}{{TP + FP}}$$

4 $$Recall = \frac{TP}{{TP + FN}}$$

5 $$F1 = \frac{2 \times Precision \times Recall}{{Precision + Recall}} = \frac{2TP}{{2TP + FP + FN}}$$

where TP (true positive) represents the number of that the sample is predicted to be positive and it is positive; TN (true negative) represents the number of that the sample is predicted to be negative and it is negative; FP (false positive) represents the number of samples predicted to be positive but being negative; FN (false negative) represents the number of samples predicted to be negative but being positive.

*Precision* measures how many of the samples predicted to be positive and they are actually positive; *Recall* measures how many of all positive samples are correctly predicted; and the *F*1 score is the harmonic mean of precision and recall, which can comprehensively consider the impact of both.

The calculation formulas for RMSE is shown in Eq. (6)^33,49^:

6 $$RMSE = \sqrt {\frac{1}{m}\sum\limits_{i = 1}^{m} {(y_{i} - \widehat{y}_{i} )}^{2} }$$

where $y_{i}$ is the real value, $\widehat{y}_{i}$ is the predicted value, and $m$ is the number of samples. RMSE reflects the difference between the predicted value and the real value. The smaller the value of RMSE, the higher the accuracy of the prediction model.

## Result and discussion

If we can freeze the lower-level parameters and only adjust the top or specific layer parameters, this will significantly reduce the number of training parameters and training time, given that the wav2vec 2.0 model has already been thoroughly trained. In addition, choosing an appropriate pooling method can effectively avoid over-fitting phenomenon. And setting an appropriate Epoch value may effectively control model convergence speed and accuracy. Therefore, we performed experiments to investigate the effect of fine-tuning or freezing on the feature encoder module parameters, the effect of different pooling methods on high-quality voice characteristics output by the wav2vec 2.0 models, and the effect of model iteration times Epoch on model convergence.

**Figure 5:**
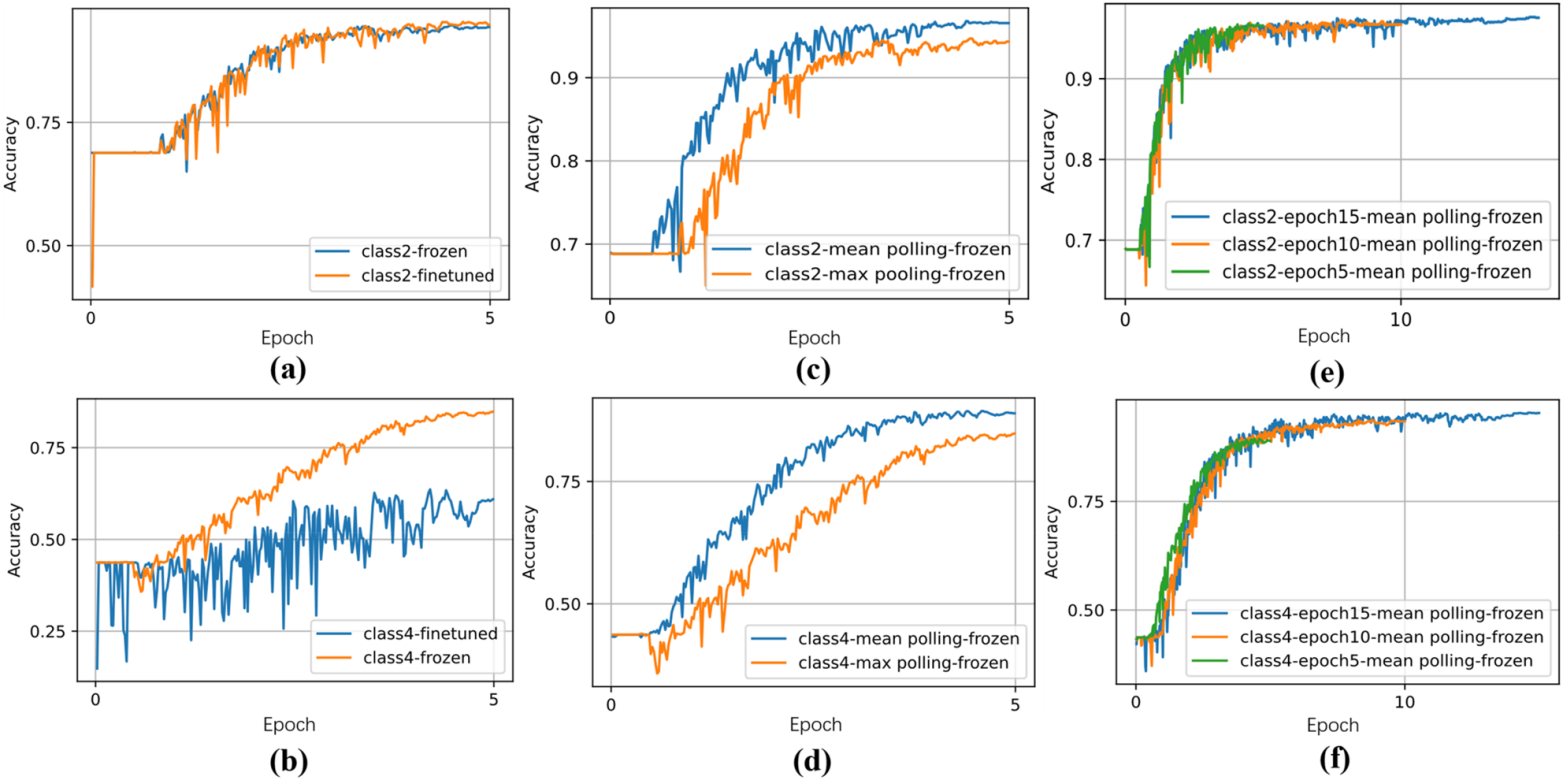
Experimental results of model fine-tuning.

In the following, “Class” is an abbreviation for “Classification”, representing the number of depression classifications. “Acc” is an abbreviation for “Accuracy”, representing the classification accuracy. In the experiment, the comparison was made between the effects of fine-tuning and freezing parameters in classification performance, Epoch size was set to 5 by prior experience. In the “Freeze” column, “True” means that the parameters of the feature encoding module in the wav2vec 2.0 model are frozen, while “False” means that the parameters of this module are fine-tuned. The specific experimental results are shown in Fig. 5a,b.

According to Fig. 5a, the accuracy of the model performance is improved by 0.45% by freezing the parameters of the wav2vec 2.0 feature encoder in the binary classification task. According to Fig. 5b, the accuracy of the model classification is substantially improved by 24.07% in the multiclassification task. Therefore, the approach of freezing the parameters of the wav2vec 2.0 feature encoder has been demonstrated to be effective. It reduces training parameters, enhances classification accuracy, while also preserving the prior knowledge of the wav2vec 2.0 model.

The study of the impact of different pooling methods on model classification performance was performed, building upon the experiment with frozen feature extractor parameters. The experimental results are presented in Fig. 5c,d. Where Max represents maximum pooling, Mean represents average pooling, and epoch size is also set to 5.

The experimental results in Fig. 5c,d demonstrate that the average pooling method outperforms the maximum pooling method. The accuracy of the average pooling method is 0.77% higher in the binary classification task, and 6.26% higher in multi-classification tasks. The effect of the epoch size on the model performance was researched at the condition of freezing the parameters and averaging pooling method. The experimental results are shown in Fig. 5e,f.

In Fig. 5e,f, it is not difficult to see that with the increase of the iteration number, the accuracy of model classification has also been improved. However, after epoch size reaches 10, the increase of the iteration number can no longer bring better effect. Comprehensively considering training time and classification performance, epoch size set to 10 is an optimal choice.

Based on the above experimental results, the best model performance is obtained, when the parameters of the feature encoder of the wav2vec 2.0 model are frozen, the average pooling method is adopted, and the epoch size is set to 10. Test set is used to evaluate the reliability of the proposed model in practical applications, and the experimental results are shown in Table 5.

**Table 5:** Performance of model classification.

Class	Label	Precision	Recall	F1	Acc
2	Dep	0.9396	0.9488	0.9442	0.9649
	Ndep	0.9765	0.9722	0.9744	
4	Non	0.9614	0.9580	0.9597	0.9481
	Mild	0.9259	0.9404	0.9331	
	Moderate	0.9589	0.9502	0.9545	
	Severe	0.9337	0.9289	0.9313	

**Figure 6:**
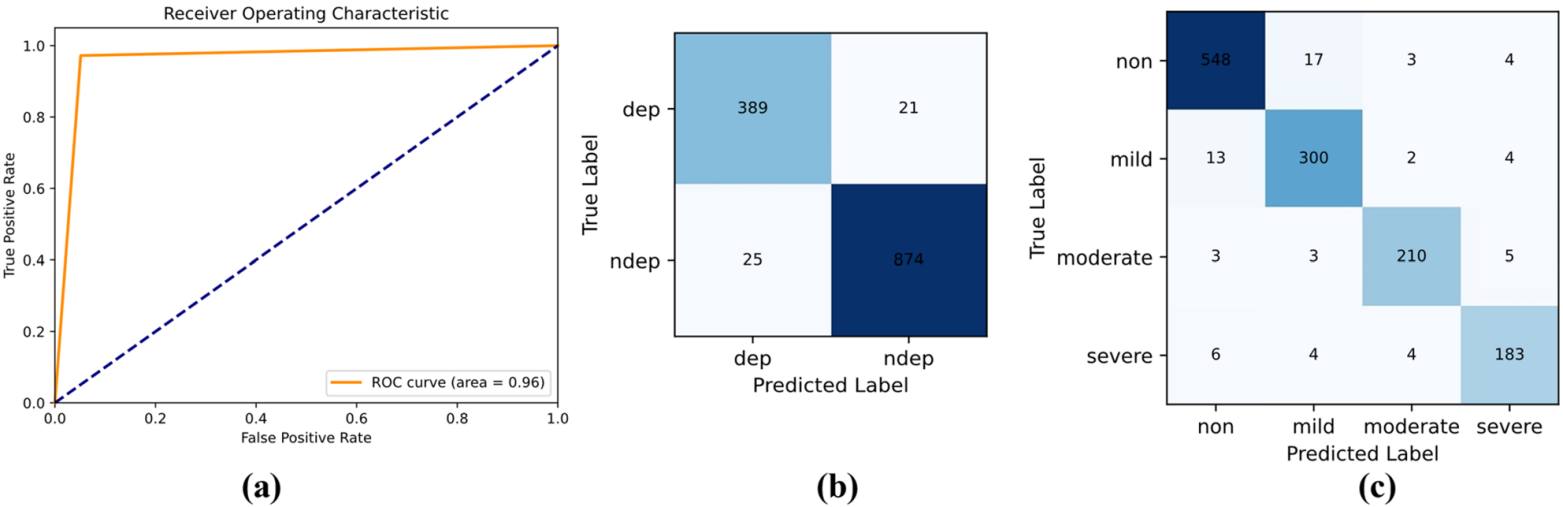
ROC and confusion matrix experimental results.

As indicated in Table 5, the model proposed in this paper demonstrates strong performance in both binary and multi-classification tasks. The overall accuracy of the binary classification task reached 0.9649, and the overall accuracy of the multi-classification task reached 0.9481. The *F*1 score reached 0.9313 and above. These show that, by use of this model, depressed individuals can be effectively identified, and the severity of their depression can be effectively evaluated. To effectively showcase the performance of the proposed model, this paper employs several evaluation metrics. For binary classification, the Receiver Operating Characteristic (ROC) curve and confusion matrix are utilized, as illustrated in Fig. 6a,b respectively. On the other hand, for multi-class classification, the evaluation is conducted using the confusion matrix, as depicted in Fig. 6c. To demonstrate that the obtained results were not due to chance, all experiments were run three times. The results of the experiments are presented in Table 6.

**Table 6:** Results of the statistical assessment.

	Mean	Std	Min	Max
Acc_class2	0.9602	0.0045	0.9541	0.9649
RMSE_class2	0.1990	0.0112	0.1875	0.2141
Acc_class4	0.9455	0.0032	0.9412	0.9481
RMSE_class4	0.4112	0.0218	0.3810	0.4317

The experimental results in Table 6 demonstrate the robustness of the model.

At present, most of the depression recognition task is a binary classification, and there are few studies on multi-classification about depression recognition. Therefore, in this paper the binary classification results are compared with the existing methods, as shown in Table 7.

**Table 7:** Comparison with existing binary classification methods.

Methods	Precision	Recall	F1	Acc
^12^	0.85	0.80	0.83	–
^13^	0.95	0.92	0.94	–
^29^	0.96 (0.95)	0.89 (0.98)	0.92 (0.97)	0.95
^33^	0.75 (0.70)	0.95 (0.26)	0.84 (0.38)	0.74
^35^	0.83	1.00	0.91	0.85
^38^	0.58 (0.67)	0.77 (0.47)	0.66 (0.55)	0.61
^39^	0.80	0.86	0.83	0.89
Ours	0.94 (0.98)	0.95 (0.97)	0.94 (0.97)	0.96

The results of identifying non-depressed individuals are shown in parentheses. According to Table 7, despite Ref.^29^ employing a multimodal fusion method, our accuracy still outperforms theirs. Specifically, the accuracy achieved by Ref.^29^ using only voice as a single modality is 0.7716, significantly lower than ours. While the Recall value of Ref.^35^ is 1.00, indicating a 100% accuracy in predicting positive samples is, our model also exhibits excellent performance with a value exceeding 0.95. Through a comprehensive comparison of multiple evaluation criteria, the performance of our model is better than that in Ref.^35^. In terms of F1 score and accuracy, our model demonstrates the best performance.

The performance of the proposed work in multi-class classification is compared with some traditional methods in Table 8. The data in Ref.^38^ is the average of the multi classification results.

**Table 8:** Comparing with existing multi-class classification methods.

Methods	RMSE	Precision	Recall	F1	Acc
^29^	3.49	–	–	–	–
^32^	4.1	–	–	–	0.8222
^34^	–	–	–	0.815	0.893
^37^	5.520	–	–	–	–
^38^	–	0.725	0.49	0.3575	–
Ours	0.3810	0.9450	0.9444	0.9447	0.9481

As shown in Table 8, the proposed model still exhibits the best performance.

## Limitations

The DAIC-WOZ dataset was constructed in a highly controlled environment and includes samples from various age groups. However, age is a factor that influences the quality of voice features, leading to noticeable discrepancy between the voice samples in the dataset and those found in the real-world scenarios. In addition, the DAIC-WOZ dataset used in the study contains only 189 samples, which is insufficient to provide adequate training. Therefore, we opted for the method of segmenting and merging voice data to augment data diversity and quantity, resulting in a total of 6545 data samples. This method avoids the effects of data enhancement introducing other noise. However, more samples still need to be included. During the experimentation process, due to limitations in computer resources, the maximum batch size for model training could only be set to 4. To address this issue, the method of gradient accumulation was employed, allowing the effective batch size to reach 8.

## Conclusions

This study proposes an effective method to enhance model performance and generalization, addressing the challenge of limited data availability. By fine-tuning the wav2vec 2.0 model on the DAIC-WOZ dataset, high-quality voice features are extracted for depression detection, resulting in a significant improvement in recognition accuracy. Notably, this approach achieves these improvements without the need for complex feature extraction, voice denoising, or data augmentation techniques. The proposed method outperforms traditional machine learning and deep learning approaches, particularly on the noisy DAIC-WOZ dataset. The study also leverages public datasets and aims to engage more volunteers in future research. However, the model’s performance with unknown data remains unverified. To enhance efficiency and applicability, additional datasets will be incorporated to further enhance the model’s robustness and generalizability.

## Data Availability

The data that support the findings of this study are available in the Institute for Creative Technologies, University of Southern California, [https://dcapswoz.ict.usc.edu/]. But restrictions apply to the availability of these data, which were used under license for the current study, and so are not publicly available.

## Code availability

The source code for all the experiments can be viewed in https://github.com/HXS572/Depression_Recognition/tree/Code.
